# In Vitro Study to Evaluate the Antibacterial Effect of an Oxidising Agent on Ex Vivo Biofilm

**DOI:** 10.3290/j.ohpd.c_2582

**Published:** 2026-03-25

**Authors:** Denise Zschach, Franca Neujahr, Paula Auschill, Anton Sculean, Christian Heumann, Nicole B. Arweiler

**Affiliations:** a Denise Zschach Dentist, Clinic of Periodontology and Peri-implant Diseases, Dental School and Hospital, Philipps University of Marburg, Germany. Writing and reviewing the manuscript.; b Franca Neujahr Dentist, Clinic of Periodontology and Peri-implant Diseases, Dental School and Hospital, Philipps University of Marburg, Germany. Conducting the study, analysing the data and reviewing the manuscript.; c Paula Auschill Student, University of Wingate, North Carolina, USA; Clinic of Periodontology and Peri-implant Diseases, Dental School and Hospital, Philipps University of Marburg, Germany. Study design, writing and reviewing the manuscript.; d Anton Sculean Dentist, Clinic of Periodontology, School of Dental Medicine, University of Berne, Switzerland. Study design, writing and reviewing the manuscript.; e Christian Heumann Statistician, Department of Statistics, Ludwig-Maximilian University, Munich, Germany. Study design, analysing the data and reviewing the manuscript.; f Nicole B. Arweiler Dentist, Clinic of Periodontology and Peri-implant Diseases, Dental School and Hospital, Philipps University of Marburg, Germany. Study design, conducting the study, analysing the data, writing and reviewing the manuscript.

**Keywords:** antibacterial mouthrinses, biofilm management, chlorhexidine, oxidising agents, plaque reduction

## Abstract

**Purpose:**

To evaluate the antibacterial effect of a mouthrinse and a fluid, both containing an oxidising agent, compared with saline (negative control) and chlorhexidine (0.2%, positive control), after application to 24-hour-old biofilms.

**Methods and Materials:**

After 24 participants had refrained from all oral hygiene measures for a period of 24 h, a voluminous biofilm sample was taken from the buccal sites of molars, smeared on a microscope slide and divided into four parts. The four samples were each coated with 5 µl of a mouth rinse solution (BMmr, blueM mouth rinsing solution, NL), a fluid (BMfl, blueM oxygen fluid, NL), chlorhexidine 0.2% (CHX) and NaCl. After 1 min, excess liquid was suctioned off, and biofilms were stained with vital fluorescent dyes for 2 min. The stained samples were covered with a cover slip, and four pictures per sample were recorded with a digital camera under the fluorescence microscope. A special image analysis program used the red and green pixels to calculate the percentage of metabolically active bacteria in the entire biofilm sample (dental biofilm vitality, VF%).

**Results:**

Both BMmr and BMfl reduced VF to 18.46 ± 9.59% and 19.53 ± 12.17% significantly (P < 0.001) compared to NaCl, with values of 59.88 ± 10.14%. CHX revealed values of 14.35 ± 6.56%, values that were not significantly lower (P < 0.001) than the other active solutions.

**Conclusion:**

Both BMmr and BMfl demonstrated a statistically significant antibacterial effect compared to NaCl and showed a similar effect to CHX. However, clinical trials are needed to evaluate the efficacy of both products containing oxidising agents when used as oral rinses.

Gingivitis and periodontitis are among the most common infectious diseases overall. There are many risk factors, including general diseases such as diabetes mellitus, but also behavioural factors such as smoking. In addition, dietary habits (high sugar consumption) and stress influence the disease, as does the presence of *A. actinomycetemcomitans* and other pathogenic germs. The effects of the disease severely restrict the affected patients in their everyday lives and have been shown to incur high treatment costs.^16,25^

A recent analysis of historically significant long-term studies on the aetiology of periodontal disease in Sri Lanka and Oslo has once again confirmed the important role that ongoing gingivitis plays in the pathogenesis of periodontitis and, in particular, attachment loss.^12^ Among other things, the authors were able to show that 99.8% of the teeth examined could be preserved for 50 years if there was no gingival inflammation, while only 63% of the teeth survived over this period if gingivitis was present.

Gingivitis and periodontitis are both triggered by dental biofilm (plaque biofilm), which is why primary prevention as well as therapy can largely be achieved through mechanical measures.^10^ However, this can be challenging for certain groups of people. While at first glance this usually brings to mind elderly or disabled people who are no longer able to perform mechanical home care tasks properly, orthodontic appliances, for example, also present young people with a major challenge. A recent review emphasises that, in addition to the breeding ground for microbial attachment that such new surface areas and crevices provide, the physicochemical interactions also contribute to altered activity in the oral microbiota and differ significantly from the symbiotic state of untreated people.^18^

Given how important it is to prevent gingivitis, home care should be supported by antibacterial agents if mechanical measures are insufficient or if certain areas cannot be reached by mechanical means. Mouthwashes for example can inactivate residual bacteria in areas that cannot be reached by toothbrushes and interdental brushes. Chemical antibacterial substances are available in various forms, such as gels, mouthwashes, and sprays, to which active ingredients are added.^9^ In the context of oral hygiene measures at home, mouthwash products can be used as adjuvants. Home antibacterial oral hygiene products can support inadequate tooth brushing by killing residual bacteria.^1,2^

There are various antibacterial agents on the market to inactivate residual biofilm. The gold standard is still chlorhexidine 0.2%.^11^ In the current international guideline on treatment of periodontitis, CHX is particularly recommended as an adjunct to subgingival instrumentation. Despite being the gold standard to support chemically mechanical measurements, CHX has some side effects such as discolouration of the teeth and oral mucosa (including the tongue) and taste irritation, so that long-term use is often not recommended.^2,7^ This challenges researchers to look for alternatives. For this reason, alternative rinsing solutions to CHX are increasingly coming into focus and are being investigated in studies, using CHX as a positive control.^1,2^

While sodium hypochlorite acting as a strong oxidising agent has been considered as an antibacterial substance for many years,^24^ in recent years a special formulation with oxidising agents has also been increasingly investigated.^5,7,8,13,17,19,21,23^ There are already positive results for anticariogenic efficacy against *S. mutans*,^21^ as well as against periopathogenic bacteria such as *A. actinomycetemcomitans* and *P. gingivalis*.^5,8^ Mattei et al (2021)^13^ were able to show in their case report that the mouthwash with this special formulation has an anti-inflammatory effect in postoperative healing.

The aim of the present *ex vivo/in vitro* study was to evaluate the antibacterial effect of oxidising agents, in the form of an oral mouthrinse and a fluid, on dental biofilm, in comparison with the gold-standard chlorhexidine and sodium chloride solution as a negative control. The study followed a design that has already been published several times by our group.^1,2,14,22^

The working hypothesis was that the antibacterial effect of both products on the *ex vivo* dental biofilm (VF in %) was not statistically significantly different to that of the negative control NaCl.

## METHODS AND MATERIALS

### Study Population

After approval by the Ethics Committee of the Medical Faculty of Philipps University of Marburg (#23-219 BO), 24 participants were recruited from the patient pool of the Clinic of Periodontology and peri-implant Diseases of Philipps University Marburg (University Clinic, UKGM). After providing written consent to participate in the study, participants were instructed to refrain from brushing their teeth for 24 h.

### Test Solutions

The following test and control solutions were used:

blueM Mouthwash fluoride-free (BMmr): manufacturer: Bluem Europe, Grote Voort 247, 8041 BL Zwolle, NL
blueM oxygen fluid (BMfl): manufacturer: Bluem Europe, Grote Voort 247, 8041 BL Zwolle, NL
Isotonic saline solution 0.9% (negative control) (NaCl): manufacturer: B.Braun Melsungen AG, D-34209 Melsungen
Chlorhexamed forte 0.2% alcohol-free (positive control) (CHX): manufacturer: GlaxoSmithKline Consumer Healthcare, Hamburg, Germany

### Evaluation of Biofilm Vitality

**Fig 1:**
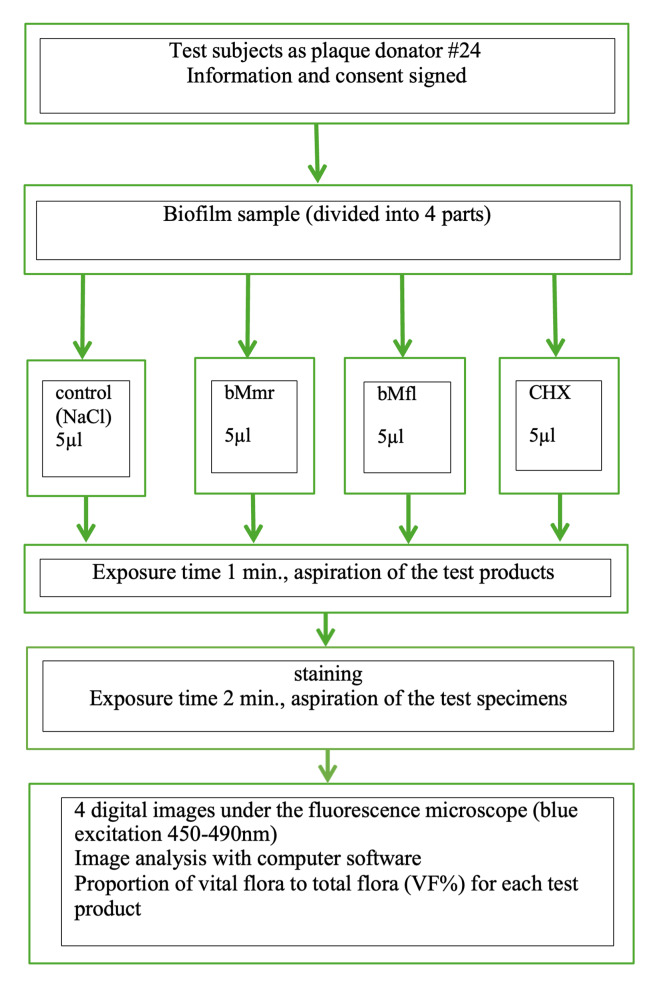
Study flow.

The study flow is shown in Figure 1. A voluminous (24-h-old) biofilm sample was taken from the molars of the participants using a sterile probe. The collected sample was smeared on a microscope slide and divided into four parts, resulting in four separate areas. Each area was covered with 5 µl of the assigned solution (CHX, BMmr, NaCl, BMfl) according to a defined scheme.

**Fig 2:**
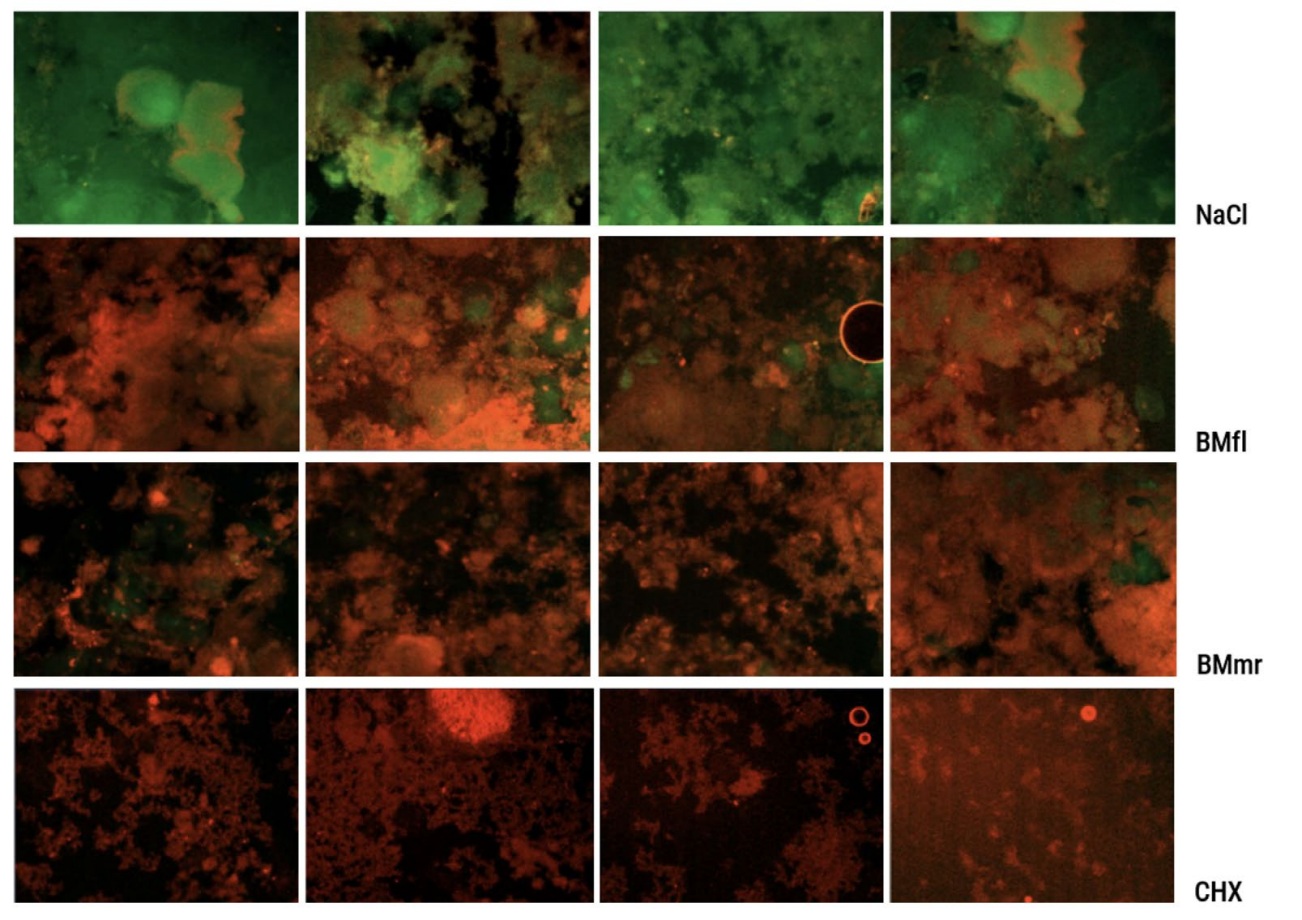
Representative microscope images stained with vital fluorescence technique after treatment with (from top to bottom) NaCl, BMfl, BMmr and CHX (vital/metabolically active bacteria are stained green, dead/metabolically inactive bacteria are stained red).

After a reaction time of 1 min, the test solutions were suctioned off, and subsequently the treated samples were vital stained according to the vitalfluorescence technique as described in detail elsewhere.^1,3,14^ Briefly, the technique is based on the use of fluorescein diacetate (FDA) and ethidium bromide (EB). FDA, a fluorescent dye, is not fluorescent itself but membrane soluble. In vital cells, it is metabolised to fluorescein, which is green and cannot leave the cell.^20^ Living or metabolically active cells are stained green (Fig 2). Dead or metabolically inactive cells are not able to metabolise the FDA, so there is no staining. A counterstaining with EB binds to the nucleic acids of dead cells and stains red. The method allows living/metabolically active and dead (metabolically inactive) cells to be simultaneously stained. Thus, a dichotomous decision: living/dead can be made for every single cell.

After a staining time of 2 min, the samples were covered with cover glass and photographed under a fluorescence microscope (Axio Imager A2; Carl Zeiss, Göttingen) to document the amount of vital bacteria in the biofilm (VF%).

A digital image analysis programme calculated the percentage of vital flora in the total flora from the number of red and green pixels (VF%).

With a digital image analysis program (ZEN 3.3, blue edition by Zeiss, Göttingen, Germany) that distinguishes between red and green pixels and calculates the proportion of green (metabolically active) bacteria in the total of red and green pixels, the percentage of vital flora in the total flora was determined as VF%.^2,14^

### Statistical Analysis

The values (VF%) of each individual image were first collected using Microsoft EXCEL and then transferred blinded to the statistician. Statistical analysis was carried out using a linear mixed model (LMM) with the study participants as a random effect (intercept, simple variance component model), the overall comparison (H0: all mean values are equal vs. at least two mean values are different, P = 0.05) was performed and since H0 was rejected, all pairwise mean comparisons were then performed (with Bonferroni correction, P = 0.05). The LMM took into account possible dependencies of the observations within a study participant.

## RESULTS

The biofilm vitality VF% of the negative control (NaCl solution) was 59.88 ± 10.14%. The positive control (CHX) statistically significantly reduced the VF% to 14.35 ± 6.56% (P < 0.001). The BMmr also achieved a statistically significant reduction in VF to 18.46 ± 9.59% (P < 0.001), which was statistically significantly lower than NaCl and only slightly higher (4.12%), but not statistically significant (P = 0.739) compared to CHX 0.2%.

The BMfl also revealed a similar and statistically significant reduction in VF% compared to NaCl (P < 0.001), which was 5.18% higher than CHX, but also not statistically significantly different (P = 0.321). The active solutions did not yield statistically significant differences from each other, while BMmr and BMfl showed similar antibacterial effects as CHX (Table 1).

**Table 1:** Results

	VF	P value NaCl	P value CHX
NaCl	59.88 ± 10.14	–	< 0.001; ***
BMfl	19.53 ± 12.17	< 0.001; ***	0.321, n.s.
BMmr	18.46 ± 9.59	< 0.001; ***	0.739, n.s.
CHX	14.35 ± 6.56	< 0.001; ***	–
Vitality (VF, in %), mean values (± standard deviation) and statistical comparison to the negative control NaCl and to CHX (n.s.: non-significant; * P ≤ 0.05; ** P ≤ 0.01; *** P ≤ 0.001).			

## DISCUSSION

The design of the present *in vitro* study was similar to that of several earlier studies performed by our group,^1,14,22^ which used biofilms directly from the oral cavity rather than mixtures of a few bacterial cultures or multispecies biofilms grown in the laboratory.

This *ex vivo* study design is an established method for investigating new microbial agents, particularly for use in the development of mouthwashes, gels, and other oral hygiene products.^2^ Even though the study was not conducted in a clinical setting, the removal of *in vivo* grown biofilms enables investigations that are much closer to the clinical situation than investigations using bacterial suspensions.

They represent an ideal study design for obtaining initial information about antibacterial properties and specific properties in comparison to CHX prior to costly clinical trials.

The fact that the active solutions did not yield statistically significant differences from each other strongly suggests that both BMmr and BMfl exhibit antibacterial effects comparable to those of CHX.

The two oxidising agents tested were a mouth rinsing solution and a fluid. It was shown that both products exhibited a statistically significant antibacterial effect in comparison with NaCl solution (negative control) and a similar effect to the gold-standard CHX (positive control). In general, the values (also for NaCl) of the study were somewhat lower compared with previous studies of the same work group.^1^ This may be due to a less established biofilm. In the present study, the biofilms were approximately 24-h old, whereas previous studies used 48-h-old biofilms. All products were tested on the same type of biofilm, ensuring consistent experimental conditions and enabling valid comparisons. Younger, 24-h-old biofilms were used because this reflects the main clinical indication for the tested products, creating a model that closely mimics real-life oral conditions.

Similar or the same oral hygiene products with the same oxidative agent have already been tested in several studies for their antibacterial effectiveness. Bali et al (2022)^4^ investigated the oxygen oxidative mouth gel in patients with moderate pocket depths during non-surgical periodontitis therapy (SRP). They were able to show that the gel achieved better results compared to the positive control with CHX (0.2%).

Furthermore, Chathoth et al (2021)^6^ were able to demonstrate antibacterial efficacy against pathogenic germs. The gel with the oxidative agent was tested by Deliberador et al (2020)^8^ in various concentrations for its effectiveness against *Porphyromonas gingivalis*. The antibacterial efficacy of the same agent in a mouth rinsing solution was also tested in regard to (specially selected) periodontal germs (*Porphyromonas gingivalis*, *Tannerella forsythia*, *Prevotella intermedia*) by Shibli et al (2021).^23^

In their study, Pawane et al (2023)^17^ tested the oxidative mouth rinsing solution in comparison to a chlorhexidine rinsing solution in the context of implant restorations. It was found that there was a statistically significant reduction in *P. gingivalis* under the oxygenated mouth rinse solution. Niveda and Kaarthikeyan et al (2020)^15^ were able to document in their split-mouth study an effect during periodontitis therapy with regard to the reduction of probing depths.

This *in vitro* study on naturally grown, mixed *ex vivo* biofilms provides a reasonable simulation of the clinical situation, as the tests were performed on biofilms formed directly on the teeth of test participants. However, *in vivo* conditions are more complex, with factors such as saliva composition, oral pH, active ingredient availability, substantivity, and surface distribution potentially modifying the effects. Clinical studies are therefore needed to confirm whether the mouthrinse, fluid, and possibly oral gel containing oxidising agents can maintain a significant antibacterial effect compared to saline and achieve efficacy comparable to chlorhexidine. To date, there have been no reports of typical side effects, such as discolouration of teeth and tongue, as well as taste irritation, as seen when using chlorhexidine. If further clinical studies confirm the efficacy of oxidising agents, they may represent a good alternative to CHX, especially for long-term use.

Future research could focus on their performance at different stages of periodontal therapy and comparisons with established benchmark solutions.

## CONCLUSION

Both BMmr and BMfl demonstrated a statistically significant antibacterial effect compared to NaCl and showed a similar effect to CHX. However, clinical trials are needed to evaluate the efficacy of both products containing oxidising agents when used as oral rinses.

### Acknowledgement

The study was partly supported by Bluem Europe, BL Zwolle, NL.

## REFERENCES
